# Editorial: Interaction between the gut flora and immunity in intestinal diseases

**DOI:** 10.3389/fimmu.2024.1458526

**Published:** 2024-08-12

**Authors:** Yating Li, Silvia Turroni, Lan Gong, Ding Shi

**Affiliations:** ^1^ State Key Laboratory for Diagnosis and Treatment of Infectious Diseases, National Clinical Research Center for Infectious Diseases, Collaborative Innovation Center for Diagnosis and Treatment of Infectious Diseases, Department of Infectious Diseases, The First Affiliated Hospital, College of Medicine, Zhejiang University School of Medicine, Hangzhou, China; ^2^ Jinan Microecological Biomedicine Shandong Laboratory, Jinan, China; ^3^ Unit of Microbiome Science and Biotechnology, Department of Pharmacy and Biotechnology, University of Bologna, Bologna, Italy; ^4^ St George & Sutherland Clinical School, Microbiome Research Centre, University of New South Wales, Sydney, NSW, Australia

**Keywords:** gut microbiota, immunity, intestinal diseases, inflammatory bowel disease, irritable bowel syndrome, colorectal cancer

It is widely recognized that the gut microbiota influences the host’s health, especially concerning immune homeostasis. The microbiome plays critical roles in the training and development of major components of innate and adaptive immune systems, with immunity regulating the equilibrium of the host-microbe relationship. Comprehending the symbiotic relationship between the gut microbiota and our immune system is crucial for both the discipline of immunology and for gaining insights into the pathogenesis of intestinal diseases. Gut microbiota disruptions are associated with a range of intestinal diseases, including immunological diseases inflammatory bowel disease (IBD) consists of two major subtypes, Crohn’s disease (CD) and ulcerative colitis (UC) (1); irritable bowel syndrome(IBS)and colorectal cancer (CRC) (2, 3).

Recently, researchers have explored microbiome-immune interactions in the development and advancement of intestinal diseases, including disease-specific bacterial lineage strains and the core microbiota in the onset and progression of intestinal diseases (1, 4, 5). The evaluation of clinical models and microbiome-immune biomarkers is predictive of the prognosis of intestinal disorders. An integrated analysis of the microbiome and metabolome uncovers distinct profiles associated with intestinal diseases (6). Metabolic characteristics are considered to act as mediators originating from the gut microbiota and are crucial in influencing the differentiation of immune cells. This is due to the capacity of bacteria to generate distinct molecules that are not produced by humans, with numerous immune cells in the intestinal tract expressing receptors for these molecules (7). The interaction between the microbiome and the immune system presents a crucial and challenging area of study, particularly in understanding the causal relationship between gut flora and the immune system in intestinal diseases. Mendelian Randomization offers a viable approach to investigating the causal relationships between microbial factors and intestinal disease (8). The management of intestinal disorders through microbiota approaches and immunotherapy strategies encompasses various interventions such as dietary modification, probiotics, synbiotics, fecal microbiota transplantation, antibiotics, prebiotics, postbiotics, modified phage therapy, and genetically engineered bacteria. Correlation studies suggest that identifying intestinal microecological regulators in clinical practice is increasingly feasible (9).

In this context, the Research Topic has proven to be relevant and has attracted considerable interest. Specifically, our research focuses on the causal link between the microbiome and immunity. Our aim is to identify the key microbiota that play a role in the development and advancement of intestinal diseases, elucidate the molecular mechanisms underlying interactions between the host immune system and the microbiome, and propose innovative pharmacological interventions targeted at intestinal diseases. The articles included in this Research Topic present original research that aligns with these objectives.

Mendelian randomization is a useful tool for exploring the causal relationships between gut microbiota and various diseases. Zhang et al. reveal that certain gut microbiota, such as phylum *Lentisphaerae*, class *Lentisphaeria*, and order *Victivallales* are associated with a lower risk of sepsis, while other microbiota, including phylum *Tenericutes* and class *Mollicutes*, are related to an increased risk of sepsis. Additionally, C-reactive protein (CRP) has been confirmed as a potential intermediate factor for the influence of the gut microbiome on sepsis. Moreover, Gao et al. identified 21 bacterial features that have a causal relationship with sepsis and its related outcomes, such as sepsis requiring intensive care and 28-day mortality. The findings of these studies contribute to the development of microbiome-based therapeutic strategies for sepsis, aimed at reducing the incidence and mortality rates of the condition.

Gut leakage and bacterial translocation are closely associated with sepsis, as well as with chronic complex disorders. Martin et al. investigate the relationship between gut barrier function and inflammation in fibromyalgia (FM) and myalgic encephalomyelitis/chronic fatigue syndrome (ME/CFS) by analyzing circulating biomarkers and self-reported symptoms. FM and ME/CFS patients had significantly higher levels of biomarkers associated with increased gut permeability (anti-beta-lactoglobulin antibodies, ZO-1) and bacterial translocation (LPS, sCD14) compared to healthy controls. Zhao et al. identify 14 specific bacterial genera associated with various peptic ulcer diseases (PUDs) types. Certain bacteria, such as *Eubacterium hallii* and *Flavonifractor*, are causally linked to esophageal ulcers, while others like *Lachnospiraceae UCG004* are associated with gastric ulcers, providing insights into the role of gut flora in PUD development. In another common gastrointestinal disorder, Wang et al. identified genetic associations between variations in gut microbiota abundance and the risk of gastroesophageal reflux disease (GERD). Specifically, the *Clostridiales Vadin BB60 group*, the genus *Lachnospiraceae UCG004*, the *Methanobrevibacterium*, and the phylum *Actinobacteria* were found to potentially have a protective effect against GERD, while the class *Mollicutes*, the genus *Anaerostipes*, and the phylum *Tenericutes* may increase the risk of GERD. Additionally, GERD was found to cause the dysregulation of 13 different gut microbiota taxa. There has been a steady growth in the number of publications in the field of gut microbiota and IBD, with a particularly significant increase in recent years, Zhang et al. conduct a bibliometric analysis of the literature in the field of gut microbiota and IBD over the past two decades. Analyzing 10,479 relevant documents from the Scopus database reveals the importance of gut microbiota in IBD research and highlights the research hotspots and frontiers in this field.

The interaction between the gut microbiota and the immune system also plays a significant role in the occurrence and development of vascular diseases. Jiang et al. demonstrate that the presence of specific gut bacteria in the host is causally associated with atherosclerosis. Different types of atherosclerosis (cerebral, coronary, and peripheral) are linked to specific gut microbiota. For example, *Ruminiclostridium* was found to have a protective effect on cerebral atherosclerosis, while *Rikenellaceae*, *Streptococcaceae*, *Paraprevotella*, and *Streptococcus* were associated with increased risk. Chen et al. investigate the causal relationship between gut microbiota and granulomatosis with polyangiitis (GPA). The study identified that one phylum, one family, and nine genera of microbiota were significantly associated with GPA, and it established that various immune cell characteristics mediated the impact of gut microbiota on GPA. For example, the family *Defluviitaleaceae* and the genus *Defluviitaleaceae UCG011* affected GPA by influencing the expression of CD11c in granulocytes.

Recently, the immunomodulatory mechanisms of some probiotics or bacterial metabolic synthesis have also attracted widespread attention. Lee et al. investigate the immunomodulatory effects of paraprobiotics derived from heat-killed *Bacillus velezensis* GV1. The study demonstrates that GV1 effectively enhances immune responses *in vitro* and *in vivo*, particularly in immunosuppressed mice treated with cyclophosphamide. Gubernatorova et al. explore the complex and controversial role of *Akkermansia muciniphila*, a mucin-degrading bacterium, in colorectal cancer. Experimental variations, such as antibiotic use, the form, or the dosage, significantly impact outcomes. The key seems to be moderation, as lower doses of A. muciniphila or its derivatives, administered without disrupting the gut microbiota, may protect against colorectal cancer. Sun et al. highlight the multifaceted role of bacterial extracellular vesicles (BEVs) in gut health and disease. BEVs, which are released by both gram-negative and gram-positive bacteria, have been traditionally viewed as waste products. However, emerging research demonstrates their significant impact on various aspects of gut homeostasis and pathogenesis.

In summary, existing evidence suggests a significant bidirectional relationship between perturbations in the microbiome and dysregulation of the immune system. The intricate communication between gut microbiota and host immunity has not been fully elucidated in the contexts of maintaining homeostasis and the progression of diseases. Therefore, comprehensive mechanistic investigations are warranted to delve deeper into the impact of microbial manipulation on host immunity and the immune response to dysbiosis of the microbiome in intestinal disorders.
